# Mechanisms of immune resistance in microsatellite-stable colorectal cancer

**DOI:** 10.3389/fonc.2026.1893907

**Published:** 2026-09-28

**Authors:** Dongguang Niu, Peng Liu, Xinyu Chen, Jingyao Li, Zhenqing Sun, Zezhou Kuang, Huichang Yu

**Affiliations:** 1 Department of Gastrointestinal Surgery, Affiliated Hospital of Qingdao University, Qingdao, Shandong, China; 2 Department of Emergency Surgery, Qingdao Geriatric Hospital, Qingdao, Shandong, China; 3 Department of Pediatric, Affiliated Hospital of Qingdao University, Qingdao, Shandong, China

**Keywords:** epigenetic regulation, immune checkpoint inhibitors, immune resistance, metabolic reprogramming, microsatellite-stable colorectal cancer, tumor microenvironment

## Abstract

Microsatellite-stable (MSS) colorectal cancer (CRC) accounts for approximately 85% of all CRC cases and exhibits limited responsiveness to immune checkpoint inhibitors (ICIs), representing a major challenge in current cancer immunotherapy. At the tumor-cell intrinsic level, low tumor mutational burden (TMB), impaired neoantigen generation, aberrant activation of oncogenic pathways such as WNT/β-catenin signaling, and defects in interferon-γ signaling contribute to impaired immune recognition and immune escape. Within the TME, the accumulation of immunosuppressive populations, including regulatory T cells (Tregs), myeloid-derived suppressor cells (MDSCs), and tumor-associated macrophages (TAMs), together with extracellular matrix remodeling and chemokine dysregulation, establishes an immune-excluded microenvironment that restricts effective antitumor immune responses. Furthermore, metabolic alterations, including enhanced glycolysis, lactate accumulation, and amino acid metabolic reprogramming, impair immune cell function and promote immunosuppression. Epigenetic modifications, including DNA methylation, histone remodeling, and non-coding RNA regulation, further contribute to the silencing of immune-related genes and reinforcement of immune evasion. In addition, recent advances in single-cell sequencing and spatial transcriptomics have provided unprecedented insights into the cellular heterogeneity and spatial organization of MSS CRC immune ecosystems. This review summarizes current knowledge regarding the molecular and cellular mechanisms driving immune resistance in MSS CRC and discusses future therapeutic opportunities for transforming immunologically “cold” MSS tumors into responsive immune microenvironments.

## 1 Introduction

Immune checkpoint inhibitors (ICIs) have revolutionized the treatment of colorectal cancer (CRC), particularly in patients with microsatellite instability-high (MSI-H)/mismatch repair-deficient (dMMR) tumors (1, 2). However, approximately 85% of CRC cases are classified as microsatellite-stable (MSS)/mismatch repair-proficient (pMMR) tumors, which exhibit limited sensitivity to ICI monotherapy, with objective response rates generally below 5% (3). MSS CRC is widely recognized as an immunologically “cold” tumor, characterized by low tumor mutational burden (TMB), insufficient neoantigen generation, impaired immune recognition, and dysregulated oncogenic pathways, including KRAS/BRAF alterations and WNT/β-catenin activation, which collectively contribute to immune exclusion and therapeutic resistance (4–6). In addition, the MSS CRC tumor microenvironment (TME) is enriched with immunosuppressive populations, including regulatory T cells (Tregs), tumor-associated macrophages (TAMs), and myeloid-derived suppressor cells (MDSCs), which suppress antitumor immunity through inhibitory cytokine production and metabolic competition (7–9).

Recent advances in single-cell sequencing, spatial transcriptomics, and multi-omics technologies have provided unprecedented insights into the complex immune landscape of MSS CRC and revealed potential strategies to overcome immune resistance (10, 11). Emerging clinical evidence suggests that rational combination approaches can partially remodel the immunosuppressive TME and enhance immunotherapy efficacy (12–14). For example, radiotherapy-based total neoadjuvant therapy combined with PD-1 blockade has demonstrated promising pathological complete response rates in patients with pMMR/MSS locally advanced rectal cancer, highlighting the potential of immune sensitization strategies (15, 16). Moreover, specific molecular alterations, including DNA damage response defects, metabolic reprogramming, and epigenetic dysregulation, have been implicated as potential determinants of immunotherapy responsiveness and therapeutic targets (17). Current strategies, including radiotherapy combinations, anti-angiogenic therapy, next-generation immune checkpoint blockade, and cytokine-based immunomodulation, are actively being explored to overcome MSS CRC immune resistance (18, 19). This review summarizes the multifaceted mechanisms underlying immune resistance in MSS CRC and discusses emerging therapeutic approaches aimed at converting immunologically “cold” tumors into immune-responsive states.

## 2 Tumor cell-intrinsic mechanisms driving immune escape in MSS CRC

### 2.1 Tumor mutational burden and neoantigen deficiency

Low tumor immunogenicity represents a fundamental mechanism underlying immune resistance in MSS CRC (20). Unlike MSI-H tumors, MSS CRC generally exhibits a low tumor mutational burden, resulting in limited production of tumor-specific neoantigens and insufficient activation of antitumor T-cell responses (21, 22). The scarcity of neoantigens represents a major barrier to effective immune recognition, as reduced antigen availability restricts the priming and expansion of tumor-reactive T-cell clones within the tumor microenvironment (23–25). Moreover, MSS CRC rarely harbors highly immunogenic mutational patterns, such as the ultramutated phenotype associated with DNA polymerase ϵ (POLE) exonuclease domain mutations, further limiting the generation of potent tumor-specific immune responses (26, 27). Beyond reduced neoantigen production, defects in antigen presentation machinery further contribute to immune escape (28, 29). Alterations affecting major histocompatibility complex class I (MHC-I) molecules, including β2-microglobulin (B2M) loss and epigenetic silencing of antigen-presentation genes, impair the ability of tumor cells to present tumor-derived antigens to cytotoxic T cells, thereby reducing immune visibility and promoting immune evasion (23, 28, 30). These mechanisms explain why strategies relying solely on immune activation, such as cancer vaccines or adoptive T-cell therapies, have shown limited efficacy in MSS CRC patients (31). Therefore, restoring tumor immunogenicity and improving antigen presentation represent critical strategies for overcoming the intrinsic immune resistance of MSS CRC.

### 2.2 Aberrant WNT/β-catenin signaling and T-cell exclusion

Aberrant activation of the WNT/β-catenin pathway is a hallmark molecular feature of MSS CRC and plays a critical role in establishing an immune-excluded tumor phenotype (32, 33). MSS CRC tumors harbor inactivating mutations in the adenomatous polyposis coli (APC) gene or activating alterations in CTNNB1, resulting in β-catenin stabilization, nuclear accumulation, and persistent activation of downstream transcriptional programs (34, 35). In addition, abnormal WNT/β-catenin signaling actively suppresses antitumor immunity by regulating chemokine expression and immune cell recruitment (36). β-catenin activation induces transcriptional programs involving factors such as activating transcription factor 3 (ATF3), which suppresses the expression of T-cell-recruiting chemokines, including CCL5 and CXCL10 (37, 38). Consequently, cytotoxic CD8^+^ T cells are excluded from the tumor parenchyma and remain restricted to peripheral stromal regions, resulting in ineffective immune surveillance (39). Genetically engineered mouse models have demonstrated that inhibition of β-catenin transcriptional activity or disruption of ATF3 restores chemokine production, enhances CD8^+^ T-cell infiltration, and improves responsiveness to immune checkpoint blockade (40). Clinically, nuclear localization of β-catenin has been proposed as a potential biomarker for predicting ICI resistance, as tumors with increased nuclear β-catenin expression exhibit significantly reduced intratumoral CD8^+^ T-cell density (41).

### 2.3 Cytokines in CRC immune resistance

Cytokines enriched within the TME represent another major factor that compromises the efficacy of tumor immunotherapy (42). Immunosuppressive cytokines can recruit, differentiate, or activate suppressive immune cell populations, thereby mediating immune escape through a dual “direct–indirect” mode of action (43). TGF-β is a multifunctional cytokine co-secreted by tumor cells, stromal cells, and platelets (44, 45). In advanced tumors, TGF-β enhances epithelial–mesenchymal transition (EMT) in CRC through the UPF2/S100 calcium-binding protein A8 axis, thereby endowing tumor cells with a migratory phenotype (46). Defects in IFN-γ signaling impair tumor cell responses to T-cell-derived IFN-γ, resulting in reduced induction of PD-L1 expression, impaired MHC expression, and decreased sensitivity to immune attack (47, 48). In parallel, TGF-β signaling directly suppresses CTL effector programs, including the expression and/or activity of perforin, granzymes, and IFN-γ, thereby impairing CD8^+^ T-cell-mediated cytotoxicity (49, 50). Notably, in MSS CRC, IFN-γ pathway dysfunction is frequently associated with increased infiltration of immunosuppressive Tregs and enhanced M2 macrophage polarization, generating a suppressive feedback network that further reinforces immune escape (51). Besides, IL-4 and IL-10, as central cytokines of the Th2-type immune response, are aberrantly overexpressed in the CRC microenvironment (52, 53). IL-10 binding to IL-10R activates steroid receptor coactivator (Src) kinase, drives the nuclear translocation of β-catenin, and enhances cancer stem cell-like properties and metastatic potential (53, 54). IL-4 induces Cyclin D1 expression in HT-29 cells through activation of the STAT6 pathway, thereby promoting tumor proliferation (55, 56). CXCL12 induces tumor cells to secrete IL-1, IL-6, and TNF-α via the CXCL12/CXCR4 axis, thereby establishing a chemokine cascade that recruits TAMs and MDSCs into the tumor parenchyma (57, 58). CXCL12 levels are also positively correlated with Tregs, and can directly activate the CXCR4–PI3K pathway in macrophage polarization, thereby promoting their proliferation and enhancing their immunosuppressive function (59) (**Figure 1**).

**Figure 1:**
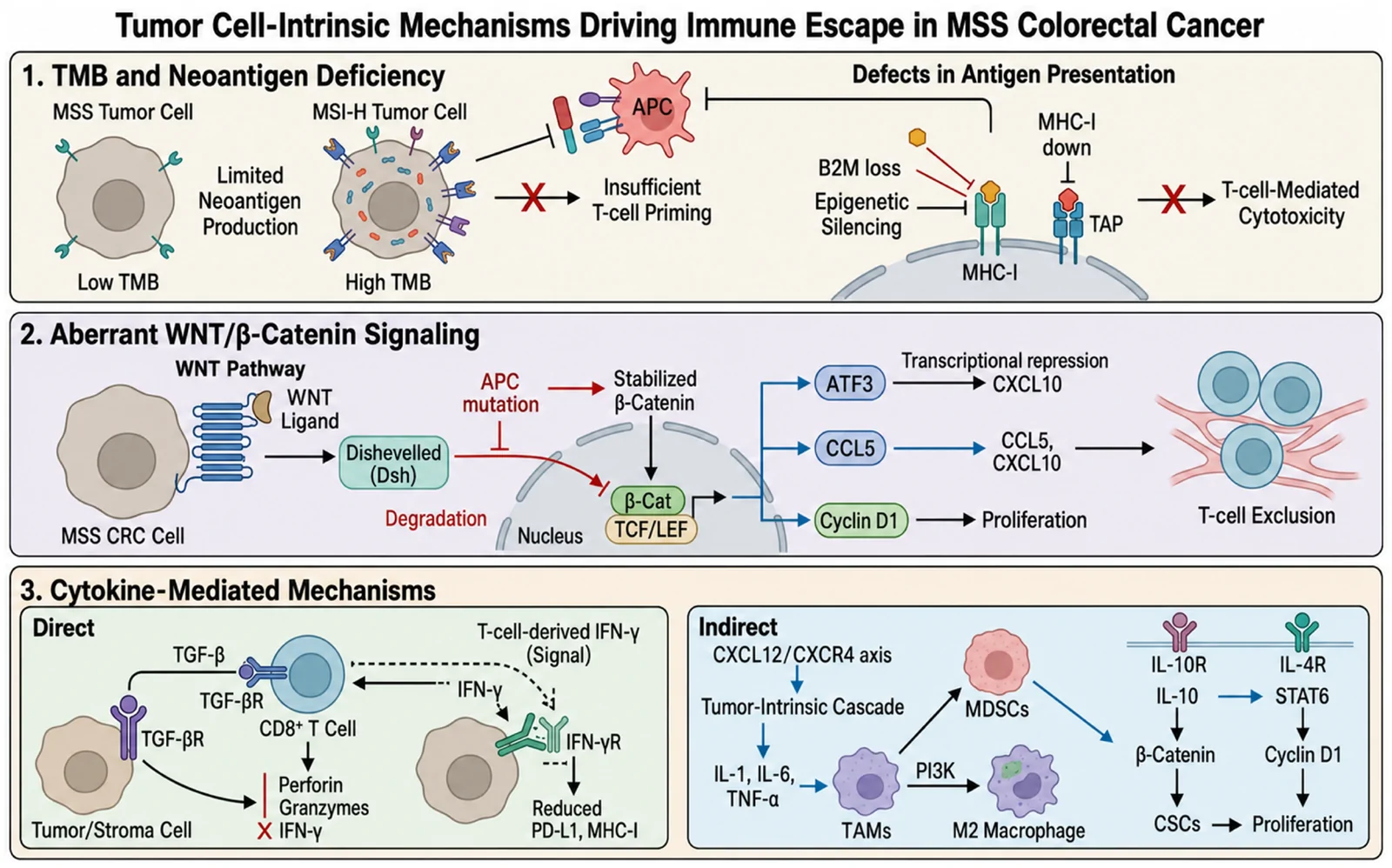
Mechanisms of immune resistance in MSS colorectal cancer.

## 3 Tumor microenvironment-mediated immunosuppressive networks in MSS CRC

### 3.1 Regulatory T cells and myeloid-derived suppressor cells

The immunosuppressive cellular landscape within the TME represents a major determinant of immune resistance in MSS CRC. Among these populations, Tregs and MDSCs play central roles in limiting effective antitumor immunity (60, 61). FOXP3^+^ Tregs are frequently enriched in MSS CRC and suppress cytotoxic immune responses through multiple mechanisms (62). These cells secrete immunosuppressive cytokines, including IL-10 and TGF-β, which inhibit CD8^+^ T-cell and natural killer (NK)-cell activation and function (63, 64). In addition, Tregs consume large amounts of IL-2 through high-affinity IL-2 receptor expression, thereby limiting the availability of this essential growth factor for effector T-cell proliferation and survival (65). Meanwhile, MDSCs, particularly the monocytic MDSC (M-MDSC) subset, accumulate within MSS CRC tumors and contribute to profound immune suppression. MDSCs inhibit T-cell activity through metabolic deprivation mechanisms, including high expression of ARG1 and inducible nitric oxide synthase (iNOS), which consume extracellular L-arginine and impair T-cell receptor (TCR) signaling by reducing CD3ζ-chain expression (66–68). Importantly, Tregs and MDSCs establish reciprocal regulatory networks that amplify immune suppression. TAMs produce CCL22 to recruit CCR4-expressing Tregs into the tumor site, while Treg-derived IL-10 further promotes macrophage polarization toward an immunosuppressive M2 phenotype (69, 70). This self-reinforcing interaction between suppressive immune populations maintains a dysfunctional immune ecosystem and contributes substantially to ICI resistance. Single-cell transcriptomic analyses have further revealed considerable heterogeneity among Treg populations in MSS CRC (10). A distinct activated Treg subset characterized by high expression of interleukin-1 receptor antagonist (IL1RN) and TNFRSF9 exhibits enhanced immunosuppressive activity and correlates with poor clinical outcomes (71, 72). These findings suggest that selective targeting of pathogenic Treg subsets, rather than broad depletion of all Tregs, may represent a more precise strategy for overcoming immune resistance.

### 3.2 Tumor-associated macrophages and cancer-associated fibroblasts as immune barriers

TAMs and cancer-associated fibroblasts (CAFs), critically contribute to immune exclusion and therapeutic resistance. TAMs in MSS CRC predominantly display an M2-like phenotype characterized by increased expression of CD163 and CD206 (73, 74). These immunosuppressive TAM populations promote immune escape through the production of TGF-β and other angiogenic and tissue-remodeling mediators, including VEGF, MMPs and SPP1, thereby promoting extracellular matrix remodeling, angiogenesis, immune exclusion, and suppression of cytotoxic T-cell activity (44, 75, 76). CAFs represent another major component of the immunosuppressive stromal network and function as key architects of immune exclusion. Activated CAFs produce abundant ECM components, including type I collagen and fibronectin, generating a dense physical barrier that prevents effective infiltration of CD8^+^ T cells into tumor nests (39, 77). In addition to their structural role, CAFs actively shape the immunosuppressive microenvironment through soluble mediators such as CXCL12 and TGF-β, thereby restricting effector T-cell infiltration and function while promoting the accumulation and/or functional polarization of immunosuppressive myeloid and regulatory immune populations (78–81). CAF-derived CXCL12 interacts with CXCR4 expressed on tumor and immune cells, contributing to tumor progression and formation of a fibrotic immune-exclusion niche (39, 82). Spatial transcriptomic analyses have provided direct evidence supporting the role of stromal organization in MSS CRC immune resistance. These studies demonstrate that CD8^+^ T cells are frequently confined to CAF-rich stromal regions and physically separated from tumor cell clusters (83, 84). Such spatial immune exclusion prevents effective tumor cell recognition and killing, even when inhibitory immune checkpoints are therapeutically blocked. Therefore, targeting CAF activation, ECM remodeling, and stromal immune barriers represents a promising strategy to enhance immune infiltration and improve ICI efficacy in MSS CRC.

### 3.3 Chemokine dysregulation and abnormal immune cell recruitment patterns

Chemokine networks play a central role in determining immune cell trafficking and spatial distribution within the MSS CRC TME. Compared with immune-inflamed MSI-H tumors, MSS CRC exhibits reduced expression of T helper 1 (Th1)-associated chemokines, including CXCL9, CXCL10, and CXCL11, which are essential for recruiting activated effector T cells into tumors (1). Conversely, immunosuppressive chemokines such as CXCL12, CXCL5, and CCL20 are frequently upregulated, promoting recruitment of suppressive immune populations while limiting cytotoxic lymphocyte infiltration (1). This imbalance generates a chemokine landscape characterized by exclusion of antitumor immune cells and enrichment of immunosuppressive populations. Epigenetic regulation represents an important mechanism underlying chemokine dysregulation. In MSS CRC, promoter hypermethylation of CXCL9 and CXCL10 suppresses their transcription, thereby impairing T-cell recruitment and contributing to immune exclusion (1). Notably, DNA methyltransferase inhibitors (DNMT inhibitors) can restore chemokine expression and enhance immune cell infiltration, providing a rationale for combining epigenetic therapy with ICIs (1). In addition, altered chemokine receptor expression further contributes to abnormal immune compartmentalization. Suppressive populations such as MDSCs and Tregs frequently express receptors including CCR5 and CXCR2, whereas effector T-cell trafficking pathways are impaired, resulting in preferential accumulation of suppressive cells within tumor regions (1). Recent studies have also highlighted the contribution of endothelial dysfunction to immune exclusion in MSS CRC. Reduced endothelial CXCL11 expression impairs T-cell extravasation into tumor tissues, while endothelial-to-mesenchymal transition (EndMT) induced by the tumor milieu further promotes stromal remodeling and immune suppression (1). Collectively, these findings indicate that immune resistance in MSS CRC is driven by a complex, multilayered disruption of chemokine signaling involving tumor cells, immune cells, and stromal components. Restoring effective immune cell recruitment and spatial organization represents an essential therapeutic goal for converting MSS CRC from an immune-excluded to an immune-responsive state (**Figure 2**).

**Figure 2:**
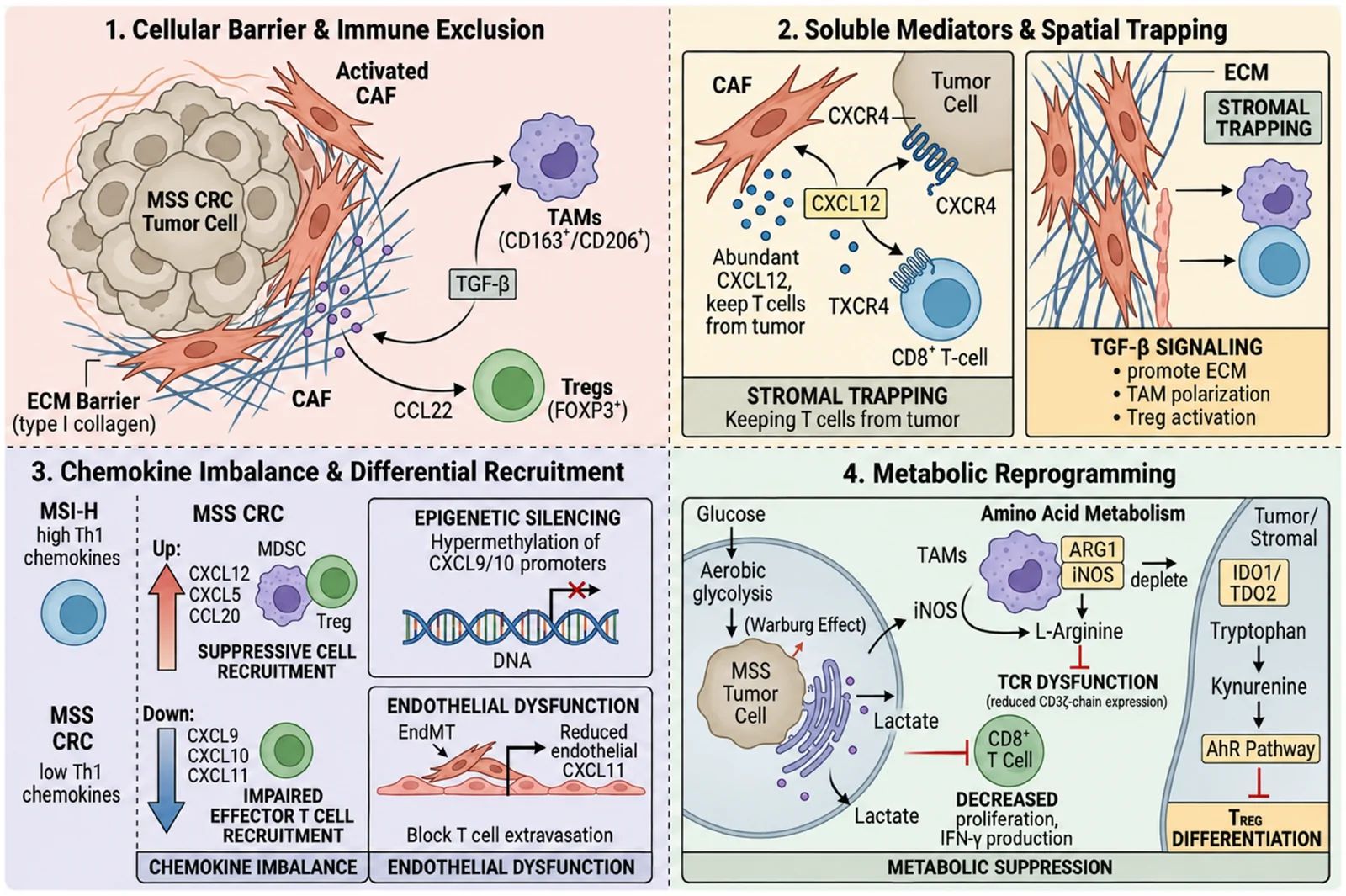
Strategies to overcome immune resistance in MSS colorectal cancer.

## 4 Metabolic reprogramming and epigenetic regulation in immune resistance

### 4.1 Tumor metabolic reprogramming and suppression of immune cell function

Metabolic reprogramming represents a critical mechanism by which MSS CRC establishes an immunosuppressive TME. Similar to many solid tumors, MSS CRC cells exhibit enhanced aerobic glycolysis (the Warburg effect), characterized by increased glucose consumption and excessive lactate production. This metabolic shift not only supports rapid tumor growth but also profoundly alters the immune landscape by creating a nutrient-deprived and acidic microenvironment. Accumulation of extracellular lactate suppresses CD8^+^ T-cell proliferation, reduces IFN-γ production, and promotes the differentiation and activation of immunosuppressive populations, including Tregs and MDSCs (17). In addition to glucose metabolism, dysregulated amino acid metabolism contributes substantially to immune escape in MSS CRC. Tumor cells and stromal components frequently exhibit increased expression of IDO1 and tryptophan 2,3-dioxygenase 2 (TDO2), leading to enhanced degradation of tryptophan into kynurenine. Kynurenine activates the aryl hydrocarbon receptor (AhR) pathway, inducing T-cell dysfunction and promoting Treg differentiation, thereby weakening antitumor immune surveillance (85). Furthermore, abnormal arginine metabolism within CAFs and TAMs contributes to immune suppression. Increased expression of ARG1 depletes extracellular L-arginine, an essential amino acid required for T-cell proliferation and cytotoxic activity. Arginine deficiency activates the GCN2 stress response pathway in T cells, resulting in impaired effector function and immune dysfunction (86). Metabolic alterations also regulate immune cell phenotypes within the TME. TAMs in MSS CRC frequently exhibit enhanced glycolytic activity, which is associated with M2-like polarization and immunosuppressive functions. Conversely, restoration of mitochondrial oxidative metabolism can promote macrophage repolarization toward an M1-like phenotype and enhance antitumor immunity (19). Therefore, targeting tumor and immune-cell metabolic dependencies represents a promising approach to remodel the MSS CRC immune microenvironment and improve responsiveness to immunotherapy.

### 4.2 DNA methylation abnormalities and silencing of immune-related genes

Epigenetic dysregulation represents another fundamental mechanism contributing to immune escape in MSS CRC. Aberrant DNA methylation frequently results in transcriptional silencing of immune-related genes involved in antigen presentation and immune cell recruitment. Specifically, promoter hypermethylation of major MHC-I genes, including HLA-A and HLA-B, as well as T-cell-recruiting chemokines such as CXCL9 and CXCL10, impairs tumor immunogenicity and limits effective immune activation (20). Genome-wide methylation profiling has revealed distinct epigenetic landscapes between MSS and MSI CRC. MSS tumors exhibit widespread CpG island methylation alterations, and specific methylation signatures, including those associated with the CpG island methylator phenotype-low (CIMP-low) subtype, are closely linked to immune dysfunction and T-cell exhaustion states (87). Dysregulated expression of DNA methyltransferases (DNMTs), particularly DNMT3A and DNMT3B, contributes to aberrant methylation of immune-associated genes. Elevated DNMT expression is negatively correlated with CD8^+^ T-cell infiltration, suggesting that DNMT-mediated epigenetic repression represents an important mechanism of immune exclusion (88). Importantly, epigenetic modifications are reversible, providing opportunities for therapeutic intervention. Preclinical studies have demonstrated that low-dose DNA methyltransferase inhibitors, such as decitabine and azacitidine, can restore the expression of chemokines and antigen-presentation genes, thereby enhancing T-cell infiltration and sensitizing MSS CRC tumors to PD-1 blockade (21). These findings have provided a strong rationale for combining epigenetic therapy with immune checkpoint inhibition as a strategy to overcome MSS CRC immune resistance.

### 4.3 Histone modifications and chromatin remodeling in immune regulation

Histone modifications and chromatin remodeling mechanisms critically regulate immune-related gene expression in MSS CRC. Histone deacetylases (HDACs), particularly HDAC1 and HDAC2, are frequently upregulated in MSS CRC and promote transcriptional repression by removing acetyl groups from histone H3 and H4 lysine residues. This process results in reduced accessibility of immune-related loci, including genes involved in chemokine production and MHC-I antigen presentation, thereby impairing CD8^+^ T-cell recruitment and activation (89). The interaction between microbiota-derived metabolites and histone regulation has further highlighted the complexity of epigenetic immune control. For example, Fusobacterium nucleatum-derived butyrate can inhibit HDAC3/8 activity and enhance H3K27 acetylation at the Tbx21 promoter, thereby promoting CD8^+^ T-cell function and improving responses to PD-1 blockade (22). This finding suggests that microbial metabolites may influence immunotherapy efficacy through epigenetic remodeling of immune cells. Enhancer regulation also contributes to immune suppression in MSS CRC. Active enhancer marks such as H3K27ac are enriched at inflammatory gene loci, including IL-6 and CCL2, promoting recruitment of TAMs and MDSCs, whereas repressive modifications such as H3K27me3 suppress enhancer activity at CXCL9/CXCL10 loci, reinforcing immune exclusion (90). In this context, enhancer of zeste homolog 2 (EZH2), the catalytic component of polycomb repressive complex 2 (PRC2), promotes H3K27me3 deposition and suppresses Th1-associated chemokines and IFN-γ response genes. Pharmacological inhibition of EZH2 has been shown to increase CD8^+^ T-cell infiltration and enhance sensitivity to PD-1 blockade in preclinical MSS CRC models (23). Chromatin remodeling complexes also contribute to immune resistance. Alterations in the SWI/SNF complex, particularly loss-of-function mutations in ARID1A, impair chromatin accessibility at IFN-γ-responsive genes and reduce tumor cell sensitivity to immune-mediated killing (91, 92). These findings establish a direct mechanistic link between chromatin remodeling defects and immune escape, highlighting epigenetic regulation as a promising therapeutic target in MSS CRC.

### 4.4 Non-coding RNA networks regulating the immune microenvironment

Non-coding RNAs (ncRNAs), including long non-coding RNAs (lncRNAs), microRNAs (miRNAs), and circular RNAs (circRNAs), represent emerging regulators of immune resistance in MSS CRC. These molecules participate in complex post-transcriptional regulatory networks that influence tumor immunogenicity, immune checkpoint expression, and immune cell function. Several lncRNAs have been implicated in immune modulation. For example, lincRNA-EPS regulates inflammatory gene expression and contributes to immune homeostasis, whereas lncRNA EGFR-AS1 promotes tumor progression through regulation of EGFR signaling and suppresses CD8^+^ T-cell-mediated cytotoxicity within the TME (93). In addition, dysregulated miRNAs contribute to immune escape by altering oncogenic signaling pathways. miR-21 promotes PD-L1 expression through inhibition of PTEN and activation of the PI3K/AKT pathway, whereas miR-93 and miR-106b regulate PTEN and BIM expression, enhancing tumor cell survival and modifying immune-related cytokine profiles (27). Circular RNAs have recently emerged as important competing endogenous RNAs (ceRNAs) involved in immune regulation. For example, circRNA_102958 regulates PD-L1 expression through miR-424 sequestration, whereas circPTEN1 influences tumor immunogenicity through regulation of PTEN expression (94). Furthermore, tumor-derived extracellular vesicles carrying ncRNAs represent a systemic mechanism of immune suppression. Exosomal miR-203 suppresses dendritic cell antigen presentation by targeting Toll-like receptor 4 (TLR4), while miR-146a and miR-155-containing vesicles contribute to immunosuppressive myeloid-cell differentiation and systemic immune tolerance (42). Collectively, ncRNA-mediated regulatory networks add an additional layer of complexity to MSS CRC immune resistance. Targeting these regulatory molecules or their downstream pathways may provide novel opportunities to remodel the immune microenvironment and enhance the efficacy of immunotherapy.

## 5 Strategies to overcome immune resistance

Overcoming immune resistance in MSS CRC requires rational combination strategies that simultaneously enhance tumor immunogenicity, remodel the immunosuppressive TME, and restore antitumor immune function. Multimodal approaches integrating chemotherapy, radiotherapy, and ICIs have shown promising results. A multicenter phase II study (NCT05839470) demonstrated that FOLFOXIRI plus bevacizumab combined with cadonilimab achieved an objective response rate (ORR) and disease control rate (DCR) of 100% in patients with pMMR/MSS CRC, although larger studies are required for validation (95). Similarly, updated data from a phase III trial indicated that penpulimab combined with bevacizumab and XELOX provided overall survival (OS) benefits and a trend toward improved progression-free survival (PFS) in metastatic CRC (96)., with consistent benefits observed in the MSS subgroup; a dedicated phase III trial in MSS mCRC is ongoing (NCT04547166) (97). In locally advanced MSS rectal cancer, the VOLTAGE-A study demonstrated that sequential nivolumab following neoadjuvant chemoradiotherapy achieved pathological complete responses (pCRs) in a subset of patients, supporting the potential of radiotherapy-induced immune sensitization (98). Consistently, a phase II trial showed that radiotherapy improved ORR and DCR following ICI treatment, suggesting that radiation may convert immune-resistant tumors into more responsive phenotypes (99).

Targeting the immunosuppressive TME represents another important strategy to overcome MSS CRC resistance. Given the plasticity of macrophage polarization, therapeutic approaches targeting TAMs include inhibition of CSF-1R-mediated M2 polarization or induction of M1-like activation through CD40 and Toll-like receptor (TLR) agonists (100, 101). Preclinical studies indicate that TAM reprogramming combined with PD-1 blockade can enhance antitumor immune responses. Other approaches, including neoantigen-based vaccines and adoptive cellular therapies such as chimeric antigen receptor T-cell (CAR-T) therapy, aim to enhance tumor-specific immune recognition (102–105). However, their clinical application in MSS CRC remains limited by tumor heterogeneity, antigen availability, and immunosuppressive TME components. Epigenetic therapies, particularly DNA methyltransferase inhibitors (DNMTis), represent additional strategies by restoring immune-related gene expression (106, 107). For example, 5-azacytidine treatment inhibited CRC cell proliferation by up to 75% *in vitro*, although DNMT inhibition alone may be insufficient to induce durable therapeutic responses, highlighting the need for rational combination approaches (108).

Metabolic modulation and dual immunotherapy have also emerged as promising approaches. In CRC models, the glutaminase inhibitor JHU-083 enhanced CD8^+^ T-cell activity, promoted antitumor immunity, and improved survival (109, 110). Targeting immune-metabolic pathways such as IDO has also attracted considerable interest, with IDO inhibitors advancing into clinical evaluation (111, 112). Furthermore, gut microbiota modulation has gained attention, as microbial species including *Akkermansia*, *Bifidobacterium*, and *Fusobacterium* have been associated with responses to anti-PD-1, anti-PD-L1, and anti-CTLA-4 therapies (113, 114). However, microbiota-based interventions require careful evaluation due to potential safety concerns. Finally, dual immunotherapy strategies are being actively investigated. In refractory MSS mCRC, the combination of botensilimab and balstilimab showed superior efficacy compared with botensilimab monotherapy in a randomized phase II study (14). However, the phase III KEYFORM-007 trial demonstrated that favezelimab plus pembrolizumab failed to improve OS compared with standard therapy in PD-L1-positive pMMR/MSS CRC, emphasizing the need for further biomarker-driven clinical studies (115).

## 6 Conclusion

Immune resistance in microsatellite-stable (MSS) CRC represents a complex and multifactorial process driven by the interplay between tumor-intrinsic alterations, immunosuppressive TME, metabolic reprogramming, and epigenetic regulation. Low tumor immunogenicity caused by limited neoantigen generation, aberrant oncogenic signaling such as WNT/β-catenin activation, and defects in interferon-γ signaling establish the foundation for immune escape. Meanwhile, the accumulation of suppressive immune populations, including regulatory T cells, myeloid-derived suppressor cells, and tumor-associated macrophages, together with stromal remodeling and chemokine dysregulation, further reinforces an immune-excluded phenotype. Emerging evidence indicates that metabolic alterations and epigenetic abnormalities are not merely secondary events but actively shape immune dysfunction by regulating immune cell activity, antigen presentation, and inflammatory signaling.

Recent advances in single-cell sequencing, spatial transcriptomics, and multi-omics technologies have provided unprecedented opportunities to dissect the heterogeneity of MSS CRC immune ecosystems and identify novel therapeutic vulnerabilities. Although current combination strategies, including radiotherapy, chemotherapy, targeted therapy, metabolic intervention, epigenetic modulation, and next-generation immunotherapies, have shown encouraging potential, durable clinical benefits remain limited and require further validation in biomarker-driven clinical trials. Future efforts should focus on developing integrated strategies that simultaneously enhance tumor immunogenicity, remodel suppressive immune niches, and restore effective immune surveillance. A deeper understanding of the dynamic interactions among tumor cells, immune populations, stromal components, and metabolic networks will be essential for transforming MSS CRC from an immunologically “cold” tumor into an immune-responsive state and ultimately achieving precision immunotherapy.
